# Factors Associated With Modern Contraceptive Use and Unmet Need for Family Planning Among Mothers in Borama, Somaliland: Community‐Based Cross‐Sectional Study

**DOI:** 10.1002/puh2.70414

**Published:** 2026-10-09

**Authors:** Nimao Hussein Ahmed, Bushra Aidarus Abdullahi, Ubah Osman Mohamed, Mustafe Mohamed Abdi, Omer Abdulkadir Osman, Abdiladiif Mawlid Muuse, Mohamed Said Hassan

**Affiliations:** ^1^ College of Health Sciences School of Medicine and Surgery Amoud University Borama Somalia

**Keywords:** Borama, family planning, maternal health, modern contraceptives, postpartum counseling, Somaliland, unmet need

## Abstract

**Background:**

National estimates in Somaliland report a modern contraceptive prevalence rate (mCPR) of 1%–3%, yet localized urban data are scarce. This study assessed mCPR, unmet need for family planning (FP), and sociodemographic determinants among mothers in Borama, Somaliland.

**Methods:**

A community‐based cross‐sectional study was conducted among 426 mothers with children under five using multistage cluster sampling (October–November 2025). Data were collected via structured interviews. Multivariate logistic regression with robust standard errors was performed to identify factors associated with contraceptive use, reported as adjusted odds ratios (AORs) with 95% confidence intervals (CIs).

**Results:**

The mCPR was 18.5%, and unmet need was 17.4%, resulting in a total demand of 35.9%. Injectables (42%) and oral pills (30%) were the most common methods. The strongest predictor of use was postpartum counseling by health workers (AOR = 18.62, *p* < 0.001) and family members (AOR = 6.30, *p* < 0.001). University education (AOR = 2.80, *p* = 0.034) and business ownership (AOR = 2.68, *p* = 0.005) also independently predicted use. Model diagnostics, including Hosmer–Lemeshow and variance inflation factor (VIF), confirmed appropriate fit and absence of significant multicollinearity.

**Conclusions:**

Contraceptive use in Borama exceeds national estimates but remains low by regional standards. Postpartum counseling serves as a critical gateway to uptake. Policy considerations may include strengthening immediate postpartum counseling and community‐based FP advocacy targeting husbands and mothers‐in‐law to address sociocultural barriers.

## Graphical abstract

Modern contraceptive use among mothers in Borama, Somaliland, was 18.5%, with 17.4% reporting unmet need for family planning. Postpartum counseling by health workers was the strongest predictor of contraceptive uptake, highlighting the need to integrate family planning counseling into routine postpartum care.

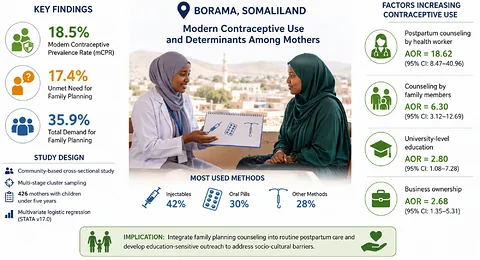

## 1 Introduction

Family planning is a key public health intervention that improves maternal health, prevents unplanned pregnancies, and reduces maternal and neonatal mortality worldwide. According to the World Health Organization (WHO), family planning allows people to attain their desired number of children and determine the spacing of pregnancies. The World Bank defines the modern contraceptive prevalence rate (mCPR), along with the WHO, as the percentage of women aged 15–49 who are married or live in union and currently use a modern method of contraception. Modern methods include female sterilization, male sterilization, the contraceptive pill, intrauterine contraceptive device (IUD), injectables, implants, female condom, male condom, diaphragm, contraceptive foam and contraceptive jelly, lactational amenorrhea method (LAM), emergency contraception, country‐specific modern methods, and other modern contraceptive methods respondent mentioned [1]. Unmet need refers to the proportion of women of reproductive age (aged 15–49 years) who are fecund and sexually active but are not using any method of contraception and report wanting to delay or stop childbearing [2].

Worldwide, the use of modern contraceptives has been increasing over the past decades. The proportion of women of reproductive age whose need for family planning is satisfied with modern methods has improved by 3.5 percentage points, increasing from 73.7% in 2000 to 77.1% in 2024 [3]. Recent global estimates indicate that more than 874 million women of reproductive age are using modern contraception [4]. Despite this progress, millions of women still face challenges accessing family planning services due to social, cultural, economic, and health‐system factors. Unmet need for family planning remains a significant issue in many low‐ and middle‐income countries, leading to unplanned pregnancies, unsafe abortions, and preventable maternal death.

Across Africa, modern contraceptive use remains relatively low compared to the global average. Sub‐Saharan Africa, in particular, has some of the lowest mCPRs globally. A recent meta‐analysis estimates the pooled MCPR in sub‐Saharan Africa to be around 22% [5]. Wide variations exist between countries, influenced by access to services, cultural norms, literacy levels, and socio‐economic conditions. Unmet need for family planning remains high in several African countries, contributing significantly to the region's high maternal mortality ratio.

Data from 17 developing countries have shown that the unmet need for postpartum family planning (PPFP) has reached 88% in some sub‐Saharan African (SSA) countries. Women in the postpartum period often have more unmet needs for modern contraceptive methods than any other group of women [6].

Ethiopia has made significant progress in its family planning programs over the past two decades, resulting in considerable increases in contraceptive use nationwide [7]. Over the last decade, the country has implemented a range of strategies to improve the uptake of modern contraceptive methods [8]. In Kenya, remarkable progress has been made, with an increase in mCPR from 53.2% in 2014 (7) to 58% in 2020 (8), although about one in ten women still have unmet need for FP [9].

Despite progress, the region still faces multiple barriers that influence contraceptive use. These barriers include limited male involvement, religious and cultural norms, inadequate health workforce, stock‐outs of contraceptive supplies, and limited knowledge about long‐acting reversible methods. Unmet need remains especially high among adolescents, rural women, and those with low levels of education [10]. The East African context demonstrates significant disparities, and challenges identified are also applicable to Somaliland. This highlights the need to generate context‐specific evidence for regions, such as Borama, to inform effective family planning programs.

Somalia and Somaliland continue to face major challenges in reproductive health, including low utilization of modern contraceptive methods and a high level of unmet need for family planning. Somalia remains among the countries with the lowest contraceptive uptake in the world. According to the Somali Health and Demographic Survey (SHDS), the mCPR is extremely low, estimated between 1% and 3%, reflecting very limited access to contraceptive services and strong social and cultural barriers to use [11]. Somalia, located in the Horn of Africa, has experienced decades of conflict and instability, which have had profound impacts on population health and development. With the majority of the population identifying as Muslim, Somali cultural and religious norms strongly shape attitudes toward sexual and reproductive health, particularly regarding contraception [12]. Unmet need for family planning in Somalia is notably high. National data indicate that approximately 37% of women of reproductive age have an unmet need for family planning, with higher levels observed in rural and nomadic communities compared to urban areas [11].

This high level illustrates that many women want to prevent pregnancy but face substantial barriers to using effective contraception. Challenges include limited availability of services, shortages of trained health providers, frequent stock‐outs of contraceptive commodities, and low levels of awareness and education regarding modern contraceptive methods [11]. Cultural and religious beliefs also play a major role in shaping attitudes toward family planning in Somalia. Community norms often favor large families, and decision‐making is frequently influenced by husbands, elders, and religious leaders [13]. Myths and misconceptions about contraceptive methods, including concerns about infertility, side effects, and perceived religious prohibition, further hinder utilization.

In Somaliland specifically, the situation mirrors the broader national trends. A study conducted in Hargeisa found that women face significant barriers accessing family planning information and services, including lack of spousal support, limited autonomy in reproductive decision‐making, and restrictive gender norms [14]. The Somaliland Health and Demographic Survey 2020 states that seven percent of the currently married women are using a form of contraception. Additionally, rural women and those with low education levels are disproportionately affected by unmet need for contraception. According to data collected in Borama, contraceptive use was low (18.5%) among 426 women, whereas 17.4% had unmet need for family planning. Overall family planning demand was 35.9%, with nearly two‐thirds having no demand or ambivalence toward contraception. These findings indicate a notable gap between family planning needs and actual contraceptive use.

## 2 Methodology

### 2.1 Study Design, Setting, and Period

A community‐based cross‐sectional study was conducted in Borama, the capital of the Awdal region in Somaliland. The study was carried out between October and November 2025. Borama is an urban center characterized by a relatively higher concentration of health facilities and educational institutions compared to the national average, which served as the context for investigating the discrepancy between local and national contraceptive prevalence rates.

### 2.2 Study Population and Eligibility

The study population consisted of all mothers of reproductive age (15–49 years) residing in Borama:

- **Inclusion criteria**: Mothers who had lived in Borama for at least 6 months and had at least one child under 5 years of age. This specific demographic was chosen because women in the first 5 years postpartum represent the highest priority group for family planning interventions to prevent short birth intervals, which are strongly linked to the region's high maternal and neonatal mortality rates.
- **Exclusion criteria**: Mothers who were critically ill or had cognitive impairments that prevented them from providing informed consent or completing the interview.

### 2.3 Sample Size Determination

The sample size was calculated using the single population proportion formula:

$$n=\frac{Z^{2}P\left(1-P\right)}{d^{2}}$$

where Z = 1.96 (95% confidence level). p = 0.5 (50% estimated prevalence was used to ensure the study was sufficiently powered, as national rates of 1%–3% would result in an unreliably small sample for multivariate analysis). d = 0.05 (margin of error).

The initial calculation yielded 384 participants. To account for a potential 10% nonresponse rate, the final sample size was set at 426 mothers. Although a design effect is often used in cluster sampling, it was not applied here because the urban population of Borama is relatively homogeneous; however, the conservative use of p=0.5 ensured a final sample large enough to maintain statistical rigor during multivariate modeling.

### 2.4 Sampling Procedure

A multistage cluster sampling technique was employed to ensure a representative sample of the urban population:

1. **Stage 1**: Borama was divided into its four primary residential zones: Sheik Ali Jauhar, Sheik Osman, Aktar, and Hayayay.
2. **Stage 2 (cluster selection)**: Within each zone, two neighborhoods (subsections) were randomly selected using a lottery method, resulting in a total of 8 clusters for the study.
3. **Stage 3 (sample allocation)**: The total sample of 426 was allocated proportionally to each of the 8 clusters based on the estimated number of households provided by local administrative records.
4. **Stage 4 (household selection)**: Systematic random sampling was used to identify households within each cluster. The sampling interval (k) was calculated by dividing the total number of households in the neighborhood by the required sample size for that cluster. The first household was selected using a random start (lottery).
5. **Stage 5 (participant selection)**: In households with more than one eligible mother, a lottery method was used to select a single participant to avoid intra‐household correlation.

### 2.5 Data Collection Tool and Quality Control

Data were collected using a structured, interviewer‐administered questionnaire adapted from the Somali Health and Demographic Survey (SHDS) and WHO family planning modules.

- **Translation**: The tool was translated into Somali and back‐translated into English by independent linguists to ensure semantic consistency.
- **Pretesting**: A pilot study was conducted on 5% of the sample (21 mothers) in the Amoud district. This was used to refine questions regarding postpartum counseling to minimize recall bias by using local terminology for health milestones.
- **Training**: Four female health professionals were trained for 2 days on interview techniques, particularly regarding the sensitive nature of reproductive health in a conservative setting.

### 2.6 Operational Definitions

Following DHS and WHO standards, the following definitions were applied:

- **mCPR**: The percentage of women currently using at least one modern method, which, per WHO/DHS definitions, includes as follows: oral contraceptive pills, IUDs, injectables, implants, female and male sterilization, emergency contraception, the LAM if within the first 6 months postpartum, and other recognized modern barrier or chemical methods.
- **Unmet need for family planning**: The proportion of women who are fecund and sexually active but are not using any method of contraception and report wanting to delay their next child for at least 2 years (spacing) or stop childbearing altogether (limiting).
- **Total demand for family planning**: The sum of women currently using contraception and those with an unmet need.

### 2.7 Data Management and Analysis

Data were entered, cleaned, and analyzed using Stata version 17.0:

- Inferential statistics: Bivariate logistic regression was performed to screen for candidate variables (*p* < 0.2). These were entered into a multivariable logistic regression model using robust standard errors to account for the multistage cluster sampling design and prevent the underestimation of standard errors due to intra‐cluster correlation.
- Model diagnostics: Multicollinearity was assessed using the variance inflation factor (VIF), with a threshold of <10. Goodness‐of‐fit was evaluated using the Hosmer–Lemeshow test. Model discrimination was assessed using the area under the ROC curve (AUC–ROC), and influential observations were screened using Cook's distance to ensure no single data point disproportionately influenced the results.

## 3 Results

### 3.1 Modern Contraceptive Prevalence

Of the 426 participants, 26.9% reported using or intending to use family planning, whereas 47.2% reported not wanting to use any method. Approximately 20.2% expressed interest but were not currently using a method, and 5.7% were unsure.

### 3.2 Family Planning Demand and Unmet Need

The study identified an unmet need for family planning of 17.4% (*n* = 74). When combined with the current use rate, the total demand for family planning in Borama was 35.9%. This indicates that approximately 51.5% of the existing demand for family planning in this community is currently being met.

### 3.3 Sociodemographic Characteristics

The majority of participants were aged 20–39 (87.1%) and were currently married (89.7%). In terms of economic status, 60.1% relied on a regular salary, whereas 24.9% owned their own business. Notably, more than half of the mothers (58.7%) reported receiving no PPFP counseling from any source (Table 1).

**TABLE 1:** Sociodemographic characteristics and family planning indicators among study participants (*n* = 426).

Variables	Frequency, *n* (%)	Contraceptive user, *n* (%)	Nonuser, *n* (%)	*χ* ^2^	*p* value
**Age group (years)**				**19.72**	**0.020**
15–19	11 (2.6)	3 (27.3)	8 (72.7)		
20–29	180 (42.3)	39 (21.7)	141 (78.3)		
30–39	191 (44.8)	35 (18.3)	156 (81.7)		
≥40	44 (10.3)	2 (4.5)	42 (95.5)		
**Income source**				**38.95**	**0.001**
Regular salary	256 (60.1)	38 (14.8)	218 (85.2)		
Own business/trade	106 (24.9)	34 (32.1)	72 (67.9)		
Remittances	33 (7.8)	7 (21.2)	26 (78.8)		
Other (casual work/none)	31 (7.2)	0 (0.0)	31 (100.0)		
**Number of living children**				**14.30**	**0.112**
1–2	141 (33.1)	33 (23.4)	108 (76.6)		
3–4	150 (35.2)	25 (16.7)	125 (83.3)		
≥5	135 (31.7)	21 (15.6)	114 (84.4)		
**Postpartum counseling**				**137.69**	**<0.001**
No counseling received	250 (58.7)	10 (4.0)	240 (96.0)		
From a family member	47 (11.0)	11 (23.4)	36 (76.6)		
From a health worker	129 (30.3)	58 (45.0)	71 (55.0)		
**Family planning demand status**				**852.00**	**<0.001**
Current user	79 (18.5)	79 (100.0)	0 (0.0)		
Unmet need	74 (17.4)	0 (0.0)	74 (100.0)		
No demand/ambivalent	273 (64.1)	0 (0.0)	273 (100.0)		

Bold values indicate statistical significance (*p* < 0.05).

### 3.4 Factors Associated With Modern Contraceptive Use

In the multivariable logistic regression analysis, PPFP counseling was the strongest factor associated with modern contraceptive use. Mothers who received PPFP counseling from a health worker had substantially higher odds of using modern contraceptives compared with those who received no counseling (AOR = 18.62; 95% CI: 8.47–40.96; *p* < 0.001). Similarly, mothers who received counseling from a family member had significantly higher odds of modern contraceptive use (AOR = 6.30; 95% CI: 2.28–17.42; *p* < 0.001).

Socio‐economic and educational factors were also significantly associated with contraceptive use. Mothers who reported owning a business or engaging in trade had higher odds of using modern contraceptives compared with those receiving a regular salary (AOR = 2.68; 95% CI: 1.34–5.35; *p* = 0.005). Likewise, mothers with university or technical education had significantly higher odds of contraceptive use compared with those with no formal education (AOR = 2.80; 95% CI: 1.08–7.26; *p* = 0.034). In contrast, primary and secondary education, remittance‐based income, husband's financial support, and age ≥40 years were not significantly associated with modern contraceptive use after adjustment (Table 2).

**TABLE 2:** Bivariate and multivariable logistic regression analysis of factors associated with contraceptive use among study participants.

Variables	COR (95% CI)	*p* value	AOR (95% CI)	*p* value
**Postpartum family planning (PPFP) counseling**				
No counseling received	Ref.	—	Ref.	—
From a family member	7.33 (2.91–18.49)	<0.001	6.30 (2.28–17.42)	<0.001
From a health worker	19.62 (9.53–40.35)	<0.001	18.62 (8.47–40.96)	<0.001
**Educational level**				
No formal education	Ref.	—	Ref.	—
Primary school	0.76 (0.34–1.72)	0.509	0.85 (0.31–2.33)	0.759
Secondary school	2.41 (1.22–4.76)	0.011	1.45 (0.59–3.53)	0.419
University/Technical education	4.74 (2.39–9.38)	<0.001	2.80 (1.08–7.26)	0.034
**Income source**				
Regular salary	Ref.	—	Ref.	—
Own business/trade	2.71 (1.59–4.62)	<0.001	2.68 (1.34–5.35)	0.005
Remittances	0.65 (0.32–1.31)	0.228	1.52 (0.52–4.50)	0.446
**Husband's financial support**				
Rarely or never	Ref.	—	Ref.	—
Most of the time	1.71 (0.73–4.02)	0.219	0.61 (0.28–1.32)	0.324
**Age group (years)**				
15–19	Ref.	—	Ref.	—
≥40	0.13 (0.02–0.89)	0.037	0.09 (0.01–1.19)	0.068

Abbreviations: AOR, adjusted odds ratio; CI, confidence interval; COR, crude odds ratio; REF, reference category.

The multivariable model demonstrated high diagnostic performance. The VIF for all included variables was below 1.8, indicating no significant multicollinearity. The Hosmer–Lemeshow test yielded a *p* value of 0.42, suggesting a good fit between the model and the observed data. The model's discrimination was also strong, with an AUC–ROC of 0.84. No influential observations were identified via Cook's distance analysis.

## 4 Discussion

This study assessed the prevalence of modern contraceptive use and unmet need for family planning among mothers in Borama, Somaliland. The findings reveal an mCPR of 18.5% and an unmet need of 17.4%. The total demand for family planning (35.9%) indicates that just over half of the demand in Borama is currently being met. Although the observed mCPR of 18.5% is significantly higher than the national estimate of 1%–3% reported in the 2020 Somali Health and Demographic Survey (SHDS), this discrepancy is largely attributable to the urban–rural divide [11]. Borama, as a major educational and commercial hub, offers better access to healthcare facilities and higher literacy rates than the rural and nomadic areas that dominate the national average. However, compared to neighboring East African urban centers in Ethiopia (mCPR ∼40%) and Kenya (mCPR ∼58%), the uptake in Borama remains low [7, 9].

One of the most significant findings was the powerful association between PPFP counseling and contraceptive use. Mothers counseled by a health worker were 18 times more likely to use modern methods (AOR = 18.62, *p* < 0.001). Although this association is exceptionally large, it must be interpreted with caution. In a cross‐sectional study, this result does not necessarily imply a purely causal relationship. It is possible that mothers with a preexisting intention to use contraceptives were more likely to seek out or remember counseling (reverse causation). Additionally, residual confounding from variables not captured in this study and measurement bias regarding the quality of counseling may have influenced this magnitude.

Education and economic status were also essential determinants. Women with university‐level education were nearly three times more likely to use contraception (AOR = 2.80, *p* = 0.034). This suggests that higher education enhances autonomy and the ability to navigate healthcare systems [8]. Furthermore, women who owned their own businesses had significantly higher odds of use (AOR = 2.68, *p* = 0.005) compared to those with regular salaries or no income. This suggests that financial autonomy may translate directly into reproductive autonomy, allowing women the independence to prioritize their health and space their pregnancies regardless of spousal or social pressure [10].

Interestingly, factors such as marital status and the number of living children did not show a statistically significant association in the multivariate analysis. In many other SSA contexts, a higher number of children is typically the primary driver for contraceptive use (limiting). In Borama, however, the fact that counseling and education were more dominant predictors suggests that the primary barrier is not a lack of desire to space births, but rather a lack of information and economic agency.

Compared to previous studies in Somaliland and the broader Somali region, the unmet need of 17.4% is notably lower than the national average of 37% [11]. This indicates a growing awareness of family planning in urban centers, yet the “gap” between demand and use remains substantial. This gap is likely driven by sociocultural barriers, including religious misconceptions and a traditional preference for large families, which continue to influence decision‐making even when services are available [13, 15].

In conclusion, service delivery factors—specifically the timing and quality of postpartum counseling—emerge as the most influential determinants of contraceptive adoption in Borama. Addressing the “information gap” through both clinical counseling and community‐based advocacy is essential to meeting the untapped demand for reproductive health services in the region.

## 5 Strengths and Limitations of the Study

### 5.1 Strengths

- **Community‐based design**: Unlike facility‐based studies, this research reached mothers in their homes across all four residential zones of Borama, reducing selection bias.
- **Robust sampling**: The use of multistage cluster sampling and systematic random sampling ensures that the findings are representative of the Borama urban population.
- **Statistical rigor**: The use of multivariate logistic regression allowed the study to isolate the independent effect of counseling and education by controlling for confounding variables, like age and marital status.
- **High response quality**: The use of female health professionals for data collection improved the accuracy of responses regarding the sensitive topic of reproductive health in a conservative cultural setting.

### 5.2 Limitations

- **Clustering and design effect**: The sample size calculation did not include a design effect, which may limit the precision of the prevalence estimates. Although robust standard errors were used in the analysis to adjust for clustering, the absence of a design effect remains a constraint.
- **Cross‐sectional interpretation**: Due to the study design, we cannot definitively establish cause‐and‐effect. The large association found for health worker counseling may be subject to reverse causation or recall bias, where recent contraceptive users provide more favorable reports of previous healthcare interactions.

## 6 Policy Considerations

The following recommendations are presented as policy considerations that require further evaluation through longitudinal or experimental research:

1. **Potential for institutionalized counseling**: Public health authorities should consider exploring the integration of structured family planning counseling during the “golden hour” after delivery.
2. **Community advocacy strategies**: It is suggested that awareness programs move beyond clinics to engage husbands and mothers‐in‐law as critical cultural gateways.
3. **Socio‐economic integration**: Development initiatives may evaluate the impact of integrating reproductive health education into women's microfinance and vocational training programs.

## 7 Conclusion and Recommendations

PPFP counseling, higher education levels, and women's economic engagement were the most significant predictors of contraceptive use. To address these findings, the following strategic actions should be evaluated:

1. **Institutionalize immediate postpartum counseling**: The Somaliland Ministry of Health may consider exploring the implementation of structured FP counseling as a routine protocol during the “golden hour” immediately following delivery and throughout the first week of postnatal care.
2. **Targeted community‐based advocacy**: Health promoters could explore adopting a “Family Influence” strategy, moving outreach beyond clinics and into homes to engage husbands and mothers‐in‐law, who act as critical cultural gateways to contraceptive use.
3. **Economic empowerment and education**: Given that financial autonomy correlates with reproductive autonomy, development NGOs should evaluate the integration of reproductive health education into microfinance and vocational training programs. Communication materials could be adapted for lower literacy populations using visual aids and Somali‐language audio content to bridge the educational gap.
4. **Expansion of choice and supply**: Health facilities should be encouraged to maintain a consistent “method mix,” including long‐acting reversible contraceptives (LARCs), to ensure that once counseling is successful, the specific demand of the mother can be immediately met.

## Author Contributions

Nimao Hussein Ahmed, Bushra Aidarus Abdullahi, and Ubah Osman Mohamed designed the study and supervised data collection. Mustafe Mohamed Abdi, Omer Abdulkadir Osman, and Abdiladiif Mawlit Muuse performed the statistical analysis and drafted the results. Mohamed Said Hassan provided technical guidance on the methodology and critically reviewed the discussion. All authors read and approved the final manuscript.

## Funding

The authors have nothing to report.

## Ethics Statement

The study was conducted in accordance with the Declaration of Helsinki. It was formally approved by the Amoud University (AU) Research Ethics Committee (REC) on September 20, 2025, and granted ethical clearance under reference number 101‐AU‐REC‐2025. The approval was valid for the duration of the data collection period (October–November 2025). Formal permission was also obtained from the Borama District Health Office.

## Consent

The authors have nothing to report.

## Conflicts of Interest

The authors declare no conflicts of interest.

## Data Availability

The datasets used and analyzed during the current study are available from the corresponding author upon reasonable request.
